# Detection Method of Fungal Spores Based on Fingerprint Characteristics of Diffraction–Polarization Images

**DOI:** 10.3390/jof9121131

**Published:** 2023-11-24

**Authors:** Yafei Wang, Xiaodong Zhang, Mohamed Farag Taha, Tianhua Chen, Ning Yang, Jiarui Zhang, Hanping Mao

**Affiliations:** 1 School of Agricultural Engineering, Jiangsu University, Zhenjiang 212013, China; wangyafei918@ujs.edu.cn (Y.W.); 1000003703@ujs.edu.cn (X.Z.); 1000006483@ujs.edu.cn (M.F.T.); 2 Department of Soil and Water Sciences, Faculty of Environmental Agricultural Sciences, Arish University, Arish 45516, North Sinai, Egypt; 3 College of Biological and Agricultural Engineering, Jilin University, Changchun 130022, China; chenth22@mails.jlu.edu.cn; 4 School of Electrical and Information Engineering, Jiangsu University, Zhenjiang 212013, China; 5 Gansu Academy of Mechanical Sciences Co., Ltd., Lanzhou 730030, China; zhangjiarui214@163.com

**Keywords:** disease spores, diffraction–polarization images, support vector machines, image processing

## Abstract

The most significant aspect of promoting greenhouse productivity is the timely monitoring of disease spores and applying proactive control measures. This paper introduces a method to classify spores of airborne disease in greenhouse crops by using fingerprint characteristics of diffraction–polarized images and machine learning. Initially, a diffraction–polarization imaging system was established, and the diffraction fingerprint images of disease spores were taken in polarization directions of 0°, 45°, 90° and 135°. Subsequently, the diffraction–polarization images were processed, wherein the fingerprint features of the spore diffraction–polarization images were extracted. Finally, a support vector machine (SVM) classification algorithm was used to classify the disease spores. The study’s results indicate that the diffraction–polarization imaging system can capture images of disease spores. Different spores all have their own unique diffraction–polarization fingerprint characteristics. The identification rates of tomato gray mold spores, cucumber downy mold spores and cucumber powdery mildew spores were 96.02%, 94.94% and 96.57%, respectively. The average identification rate of spores was 95.85%. This study can provide a research basis for the identification and classification of disease spores.

## 1. Introduction

There has been a growing demand for “vegetable basket” projects in recent years due to individuals’ aspirations for an improved quality of life [1,2]. Currently, China boasts a protected cultivation area of over 4.2 million hectares, extensively dispersed throughout the country, securing its position as the global leader in this domain [3,4]. The vegetable basket project in China is widely regarded as a crucial pillar of support and a significant avenue for farmers to enhance their revenue [5]. Tomatoes and cucumbers enjoy considerable popularity among customers because of their flavorful profiles and high nutritional content. Moreover, the cultivation space dedicated to these crops constitutes a substantial portion of China’s protected agriculture area [6,7]. The prevailing temperature and humidity conditions within the greenhouse are conducive to the occurrence and transmission of airborne fungal infections. Examples of such diseases are the gray mold of tomatoes, the downy mildew of cucumbers and the powdery mildew of cucumbers [8,9]. The prevalence of airborne fungal diseases is expected to rise annually due to the growth of cultivated areas and the prolonged practice of continuous cropping. Consequently, this trend may significantly reduce crop yield, ranging from 20% to 50%, and in some cases, complete crop failure [10]. In addition, because airborne fungal spores travel through the air with air currents, people working in greenhouses may experience allergic reactions if they inhale these fungal spores. Hence, timely detection and prevention of airborne diseases in greenhouses are essential.

Traditional detection techniques for disease spores mainly include electron microscope detection, polymerase chain reaction, biological detection, etc. Despite their ability to accurately detect disease spores, these approaches encounter several challenges, including technical requirements, prerequisites, intricate procedures and limited accessibility [11]. With the technological revolution, relevant scientists have researched the classification and recognition of disease spores based on machine learning and image processing. For example, Lei et al. [12,13] used a portable spore catcher to collect urediniospores. Images of urediniospores were taken with a microscope, then the urediniospores’ image was processed via threshold segmentation, contour extraction and morphological manipulation. A remote monitoring platform of urediniospores was built to collect and count wheat stripe rust spores in real time. In their study, Yang et al. [14] utilized a mix of texture and shape features and a decision tree confusion matrix approach to distinguish between rice false smut and blast. This distinction was made based on the analysis of microscopic spore images. Additionally, the researchers advocated using the distance transformation–Gaussian filtering algorithm as part of their methodology. After separating the spores, a decision tree model classification was employed, utilizing four shape and three texture features. The resulting detection accuracy was found to be as high as 94%. Although the above method can detect the disease spores, it is difficult to capture the complete spore image because of the small field of view of the traditional microscopic imaging technology, and it is easy to cause large errors.

Researchers have recently preferred holographic imaging technology due to its notable advantages, including a wide imaging field of view and cost-effectiveness. Luo et al. [15] measured target analyte concentration in quantitative particle agglutination tests based on mobile lensless microscopy and deep learning. Using diffraction fingerprint feature processing, Wang et al. [16] selected thirteen diffraction fingerprint features to classify fungal spores. The classification results for micro-particles based on diffraction fingerprint features could be better due to the small number of extracted fingerprint features. One additional characteristic of light is its polarization, which possesses a distinct benefit not found in regular image and reflection spectra. It can convey some information that is challenging to characterize using intensity images and spectra [17]. Extracting the feature information of objects in different polarization directions and fusing it can improve the recognition rate of objects [18,19]. For example, Jiang et al. [20] studied the polarization–diffraction imaging method for the accurate classification of malignant and benign tumors. Extracting morphology-related “fingerprints” can significantly improve the diagnosis and early warning of tumors. Therefore, diffraction and polarization imaging techniques can be combined. The classification and recognition of disease spores were carried out based on the diffraction–polarization fingerprint image features.

This study introduces an approach for classifying airborne disease spores in greenhouse crops, utilizing the distinctive fingerprint traits observed in diffraction–polarization images. Initially, the diffraction–polarization imaging system of disease spores was constructed to capture spore diffraction–polarization images. Subsequently, the diffraction–polarization images were processed, wherein the fingerprint features of spore diffraction–polarization images were extracted. Finally, an SVM classification algorithm was used to classify the disease spores. This study introduces a method to classify spores by using fingerprint characteristics of diffraction–polarization images and machine learning. This study provides a research basis for the identification and classification of disease spores.

## 2. Materials and Methods

### 2.1. Sample Preparation

Cucumber and tomato plants were cultivated in a Venlo-type greenhouse at Jiangsu University, Zhenjiang, China. The greenhouse was oriented east-west, with a top height of 4.73 m and a shoulder height of 4.0 m; each span was 3.2 m, and the length of the greenhouse measured 45 m. The greenhouse had an insulation curtain, the use of pipeline heating, a sunshade net, a wet curtain fan and other cooling equipment, and environmental control using computer-automated control system regulation. In order to obtain the disease spore samples, tomato gray mold spores were collected when gray mold occurred in tomato plants. The tomato cultivar of “Zhejiang Powder 202” (Zhejiang Yinong Seed Industry Co., Ltd., Hangzhou, China) and the cucumber cultivar of “Jinyou No. 1” (developed by Tianjin Academy of Agricultural Sciences) were used to conduct the field experiments. To procure a sample of crop disease spores, the application of pesticides was withheld during the planting process. Fresh diseased leaves were collected from infected cucumber plants after natural disease. Individual cucumber downy mildew and powdery mildew spots with sufficient incidence and distance from other spots were cut with scissors. They were then dipped in sterile water, spotted down, and gently applied to pre-planted cucumber leaves [21]. Due to the inability to develop cucumber downy mildew spores and cucumber powdery mildew spores in vitro, it was necessary to transfer these spores from previously diseased cucumber plants to freshly cultivated cucumber plants to preserve the samples, and also to achieve the purpose of expanding propagation and culture [22]. Tomato gray mold spores can be cultured in vitro. To obtain an uncontaminated sample of tomato gray mold spores, a leaf with diseased spots was first cut from an infected tomato plant, dipped in sterile water, and then attached to a non-infected tomato plant with the diseased spots facing downwards. This was repeated until gray mold was the only spot on the tomato plant. Then, tomato leaves with gray mold were placed in a PDA (Potato Dextrose Agar) medium. Subsequently, the preservation and propagation of the strains were carried out [23]. In order to determine whether the prepared pathogen spores were the desired target fungal spores, cucumber and tomato plants were infected in a closed space. It then was observed whether the cucumber plants developed mildew and downy mildew and the tomato plants developed gray mold. If the cucumber plants developed powdery mildew and downy mildew and the tomato plants developed gray mold, then it confirmed that the prepared pathogen spores were the target fungal spores needed for the research.

### 2.2. Diffraction–Polarization Fingerprint Image Acquisition System

**Figure 1:**
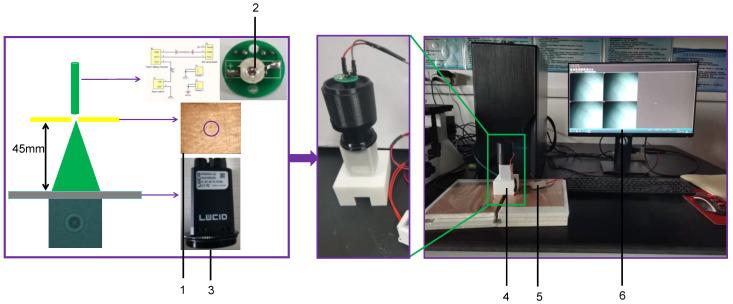
Diffraction–polarization fingerprint image acquisition system. 1. Copper plate with 100 μm micropore. 2. LED lamp beads with a central wavelength of 435 nm. 3. Polarization camera. 4. Diffraction–polarization device. 5. Power Supply. 6. Computer.

Traditional low-light imaging technology means that visible light can be transmitted or reflected through the sample, and, after passing one or more lenses, a magnified image of a tiny sample can be obtained [24]. Diffraction refers to the phenomenon where light waves deviate from a straight line and propagate behind obstacles when they encounter obstacles or holes in the propagation process. By observing the light and dark areas that appear on the screen, the distribution of light-intensity can reflect the imaging information of the object [25]. Polarization imaging technology can detect the polarization information of the object surface, and compared with ordinary optical images, the brightness and contrast of the object and the background in the polarization image are relatively enhanced [26,27]. Therefore, this study combined the characteristics of diffraction imaging and polarization imaging to build a fungal spore diffraction–polarization fingerprint image acquisition system (Figure 1).

As shown in Figure 1, the device structure of the diffraction–polarization fingerprint image acquisition system was designed using Solid Works 2020 software, and the shell was printed via 3D printing technology. In order to eliminate the interference of external light, the color of the 3D printing material in the diffraction device was black. The color of the 3D printed material for the polarizing camera support frame was white. Select LED lamp beads with a central wavelength of 435 nm (the actual wavelength was 430–440 nm) as the light source were used. However, as the light source of the diffraction–polarization imaging system, it needed a power supply circuit and a circuit to adjust the brightness of the light source. Therefore, this study used Altium designer 2019 software to draw the circuit diagram of the light source and import it into the PCB diagram, then make the PCB circuit board, and finally, weld it into the light source. The system used a 5 V power supply to power the light source. The diameter of the micropore was 100 μm. The microhole was directly below and next to the light source. The polarization camera was a TRIO5OS-QC model (purchased from LUCID Vision Labs, Richmond, BC, Canada). The polarizing camera was located 45 mm directly below the micropore. The polarizing camera was connected to the computer via a data cable. The computer was equipped with the diffraction–polarization fingerprint image acquisition software Arena View-Arena View2.

### 2.3. Methods of Collecting Fungal Spores

**Figure 2:**
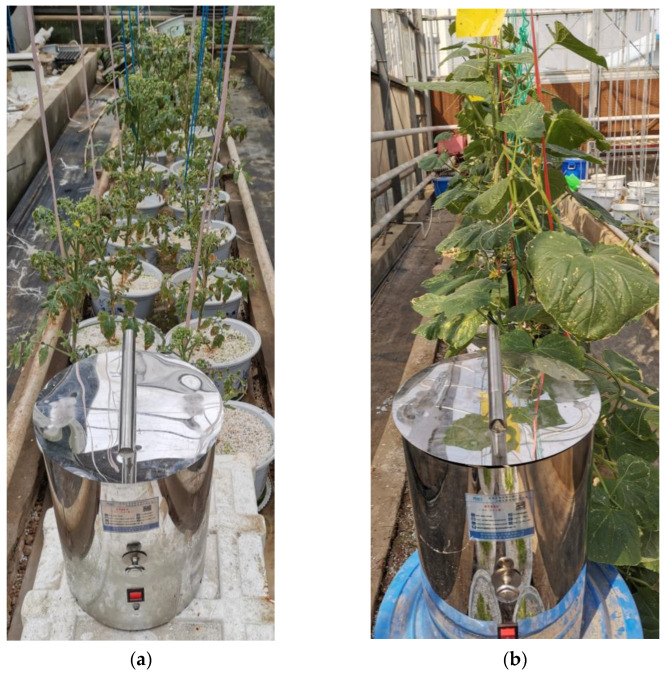
Collection of fungal spores. (**a**) Collection of fungus spores from tomato gray mold. (**b**) Collection of fungus spores from cucumber powdery mildew and downy mildew.

In order to collect the diffraction–polarization fingerprint of fungal spores, tomato and cucumber plants were cultivated in a greenhouse. In the flowering stage of tomato plants, tomato gray mold spores were prepared in a spore suspension of 1 × 10^6^ spores/mL with sterile water, and the pathogen was inoculated by leaf spraying [28]. While the cucumber plants were in bloom, the cultivated cucumber downy mildew leaf and the cucumber powdery mildew leaf were dipped into the newly cultivated cucumber plants. After the tomato and cucumber plants were diseased, a portable spore catcher was used to collect fungal spores in the air, as shown in Figure 2.

### 2.4. Collection of Diffraction–Polarization Fingerprint Images for Fungal Spores

Diffraction–polarization images of spores were taken at the bioinformatics analysis laboratory of the College of Agricultural Engineering, Jiangsu University. Firstly, the slides in the spore catcher were taken out and then looked at under a microscope to determine if there was a target fungal spore. Secondly, the slides containing target fungal spores were placed into the diffraction–polarization fingerprint image acquisition system. Then, the computer and the diffraction–polarization fingerprint image acquisition software Arena View-Arena View2 were started up. The connected polarization camera model in the diffraction–polarization fingerprint image acquisition software was selected. The image acquisition mode for polarized images in the directions of 0°, 45°, 90° and 135° was selected. Finally, polarization fingerprint images of the fungal spores were taken.

### 2.5. Collection and Processing of Spore Diffraction–Polarization Images

**Figure 3:**
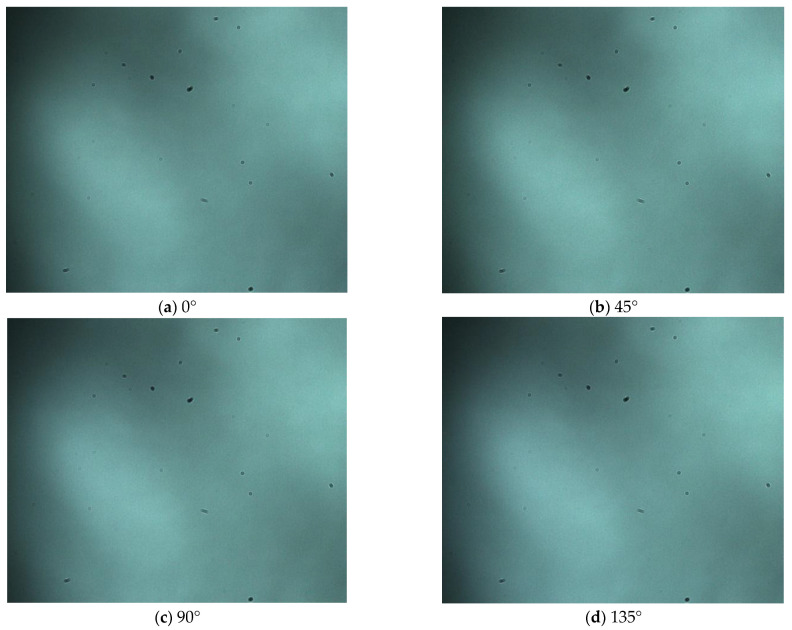
Diffraction–polarization fingerprint image of disease spores.

In this study, the polarization images for diffraction fingerprints of disease spores in the directions of 0°, 45°, 90°and 135° were collected. The collection process of diffraction–polarization images of airborne disease spores was as follows: firstly, the airborne disease spores were captured in the greenhouse with a portable spore capture instrument, and the glass slide loaded with spores was put into a closed box and taken to the laboratory. Then, the glass slide loaded with airborne disease spores was put on the diffraction–polarization image collection system. Finally, the diffraction–polarization image collection system was turned on and the diffraction–polarization images of spores were taken. A spore diffraction–polarization fingerprint image is shown in Figure 3.

The acquisition system for diffraction–polarization fingerprint images of spores is susceptible to environmental influences throughout its operational procedures. Hence, it becomes imperative to pre-process the acquired image to mitigate the presence of extraneous data in the image of the airborne disease spore diffraction–polarization fingerprint and preserve pertinent information. The specific steps were as follows:

Initially, the diffraction–polarization fingerprint image of spores was processed via two-dimensional gamma function. The expression for the two-dimensional gamma function is provided [29]:

(1) $$O(x,y)=255{\left(\frac{F(x,y)}{255}\right)}^{\gamma }$$

(2) $$\gamma ={\left(\frac{1}{2}\right)}^{\frac{m-I(x,y)}{m}}$$

where, *O*(*x*, *y*) represents the brightness value of the output image after correction; *F*(*x*, *y*) represents the original image of the input; *γ* represents the brightness enhancement index value, which includes the light component characteristics of the image; *I*(*x*, *y*) represents the extracted light component; and *m* represents the mean brightness of the light component.

Secondly, to maintain the intricate characteristics of airborne disease spores, it is imperative to mitigate the presence of noise in the image. Median filtering is nonlinear and effectively addresses impulse noise. Additionally, it effectively preserves the edge information of the image. Thirdly, after removing this noise, the OTSU method was used to automatically find the threshold of the diffraction–polarization fingerprint image of airborne disease spores. Then, the target image was obtained through morphological operation, smoothing and hole filling.

**Figure 4:**
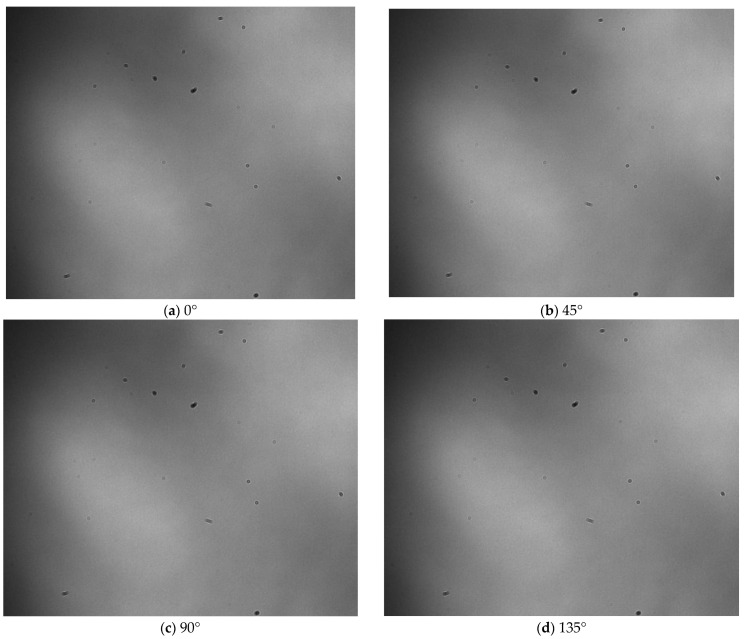
Diffraction–polarization fingerprint image of airborne disease spores after gray processing.

Furthermore, due to using a color camera in the polarization, the acquired diffraction–polarization images of airborne disease spores possess specific color-related data. To obtain the fingerprint features of diffraction–polarization images of airborne disease spores, it is important to convert color images into grayscale images. Figure 4 displays the diffraction–polarization image of airborne disease spores after processing.

**Figure 5:**
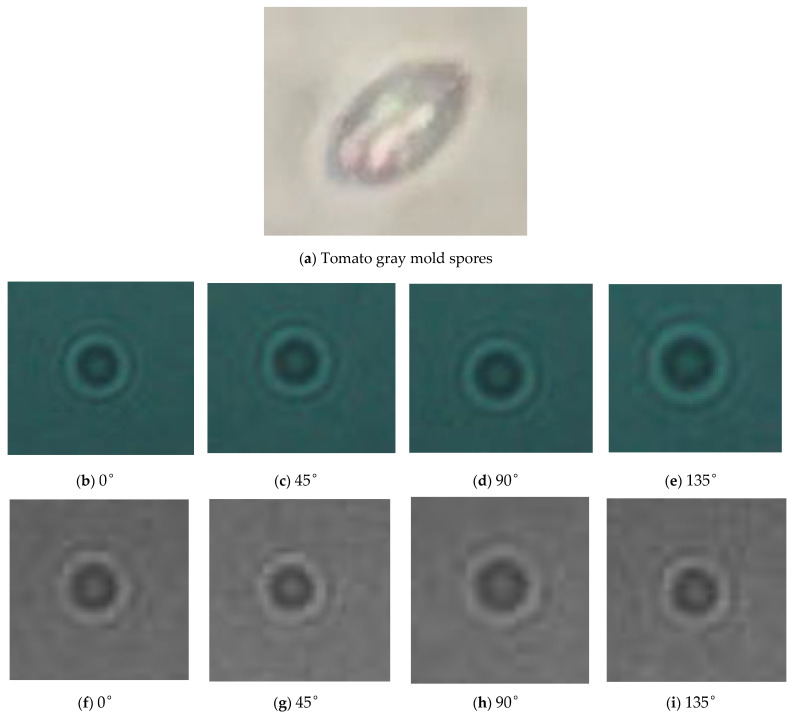
Fungal spores and diffraction–polarization fingerprint images.

Figure 5 shows the spore image of tomato gray mold spores and its corresponding diffraction–polarization fingerprint image.

### 2.6. Feature Extraction of Airborne Disease Spore Diffraction–Polarization Fingerprint Image

The extraction process of the relative light-intensity distribution of the airborne disease spore diffraction–polarization fingerprint image was as follows:

① The preprocessed diffraction–polarization fingerprint image of airborne disease spores was saved as a file in .bmp format;

② The diffraction–polarization fingerprint image of airborne disease spores saved in .bmp format was imported into MATLAB software R2016b by using import data;

③ The format of the imported image data was converted into a double-precision data type by using the double () instruction;

④ The mesh () instruction was used to generate double-precision image data into 3D images;

⑤ The contour () command was used to extract a contour map of the 3D image;

⑥ A two-dimensional section of contour map was extracted.

**Figure 6:**
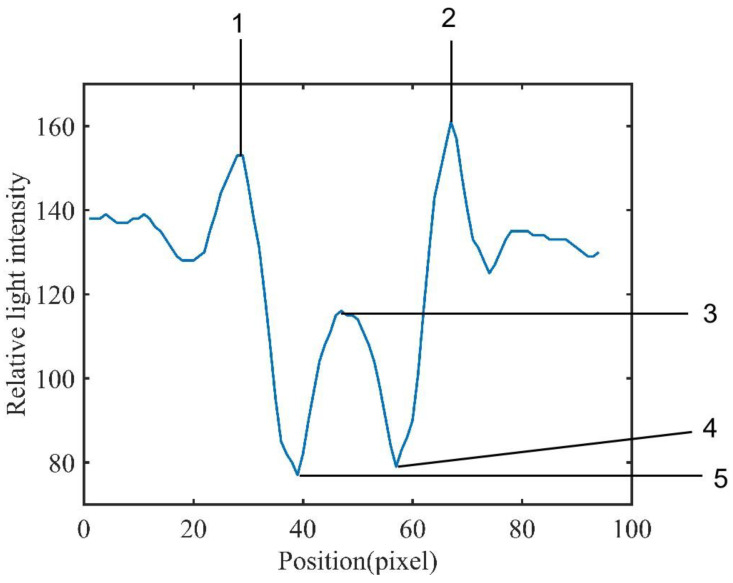
Characteristics of disease spores diffraction–polarization fingerprint images: 1 and 2, main bright fringe (P); 3, center (C); 4 and 5, main dark fringe (V).

The characteristics of the diffraction–polarization fingerprint of airborne disease spores are closely related to the species, size, geometry and light absorption of spores. According to the extraction process of the airborne disease spore diffraction–polarization fingerprint image, the relative light-intensity distribution of the airborne disease spore diffraction–polarization fingerprint was obtained, as shown in Figure 6.

It can be seen from Figure 6 that the pixel distribution in the area near the center of the diffraction fingerprint contributes the most to the eigenvalue. Therefore, the main bright fringe (P), center (C) and main dark fringe (V) of the airborne disease spores diffraction–polarization fingerprint was selected. Five diffraction fingerprint features were selected to classify airborne disease spores (Figure 6). These five eigenvalues are divided into three categories: they are the main bright, dark, and center edges. Then, these three types of features were calculated. The ratios of peak to center (PCR), valley to center (VCR), and peak to valley (PVR) were obtained. The calculation formula was as follows:

(3) $$PCR=\frac{{AP}_{i}}{C}\;(i=1,2)$$

(4) $$VCR=\frac{{AV}_{j}}{C}\;(j=1,2)$$

(5) $$PVR=\frac{{AP}_{i}}{{AV}_{j}}\;(i=1,2;\;j=1,2)$$

where, $AP_{i}$ represents the peak of the airborne disease spores diffraction–polarization fingerprint; $AV_{j}$ represents the trough of the airborne disease spores diffraction–polarization fingerprint; and C represents the central area of the airborne disease spores diffraction–polarization fingerprint.

### 2.7. Evaluation Index

In machine learning, a confusion matrix is a standard format for expressing accuracy evaluation [30]. In this study, a confusion matrix was used to evaluate the classifier’s performance. In addition, the calculation formula of the evaluation index for classification effect was as follows [31]:

(6) $$Accuracy=\frac{TP+TN}{TP+TN+FP+FN}$$

(7) $$Precision=\frac{TP}{TP+FP}$$

(8) $$Recall=\frac{TP}{TP+FN}$$

(9) $$F1-Score=\frac{2\times Precision\times Recall}{Precision+Recall}$$

where, *TP* (True Positive) represents positive samples predicted as positive; *FP* (False Positive) represents negative samples predicted as positive; *FN* (False Negative) represents positive samples predicted as negative; *TN* (True Negative) represents negative samples predicted as negative.

When performing the classification, the actual predicted spore number of airborne diseases was regarded as a positive sample number, and the sum of spore numbers of other airborne diseases was a negative sample number.

### 2.8. Statistical Analysis Software

In this study, pathogenic spore diffraction was processed using MATLAB R2016b software. All algorithms were run in a MATLAB R2016b environment.

## 3. Results and Discussion

### 3.1. Results for Feature Extraction

**Figure 7:**
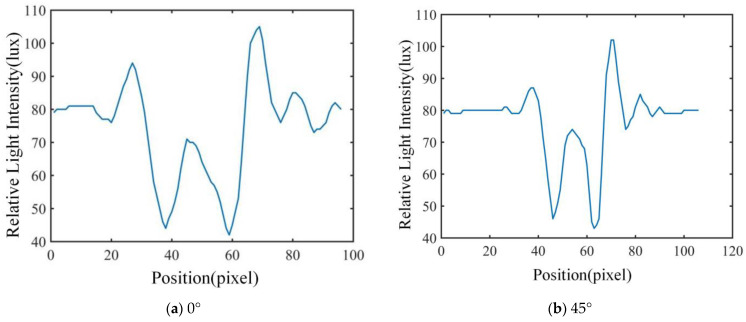

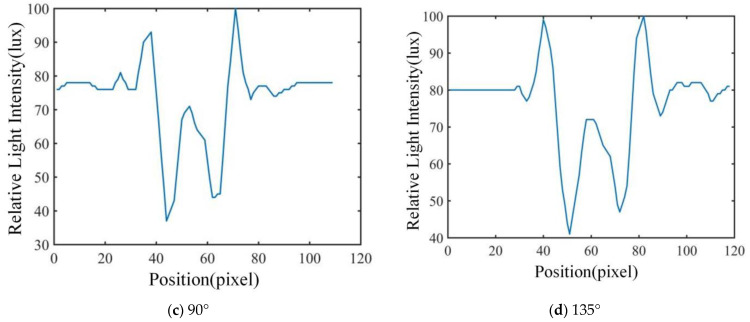
Cucumber powdery mildew spore characteristic extraction results for diffraction–polarization fingerprint image.

**Figure 8:**
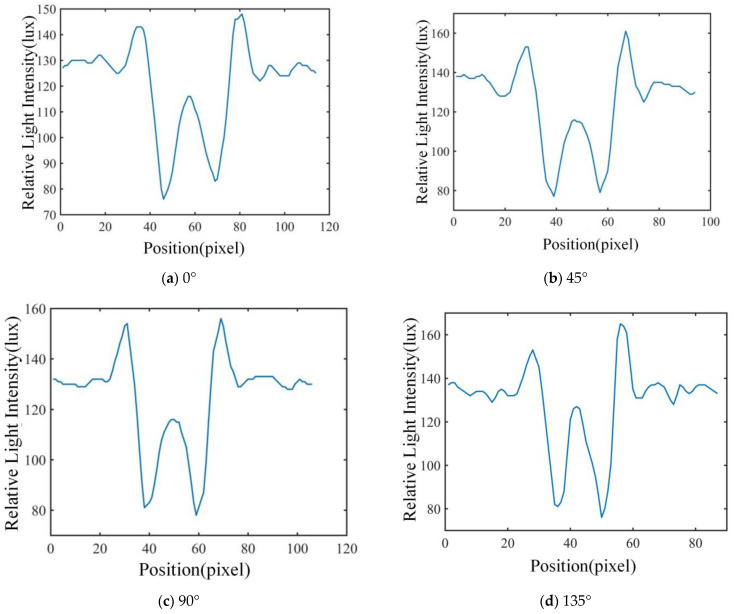
Tomato gray mold spore characteristic extraction results for diffraction–polarization fingerprint image.

**Figure 9:**
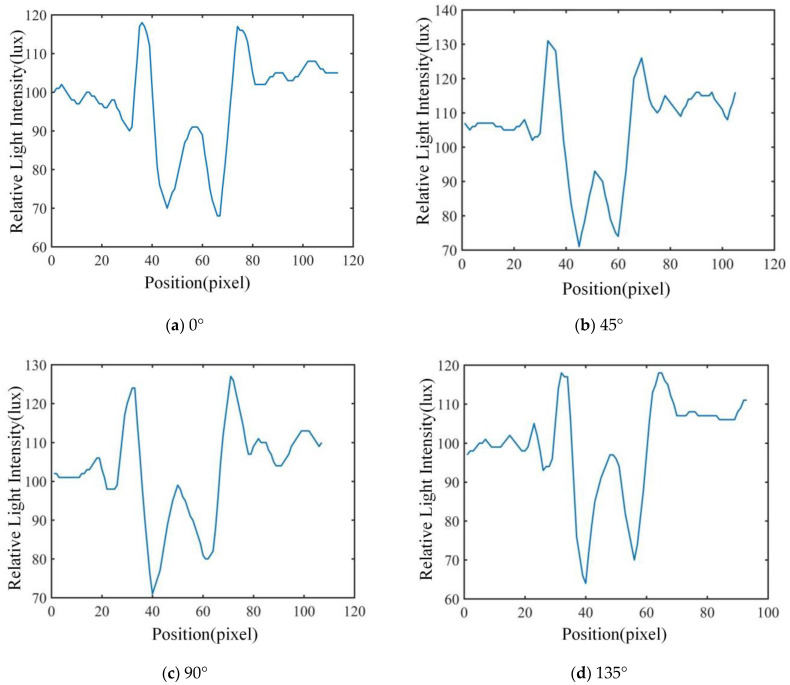
Cucumber downy mildew spore characteristic extraction results for diffraction–polarization fingerprint image.

In this study, the polarization images of the airborne disease spore diffraction fingerprints in 0°, 45°, 90° and 135° directions were obtained, respectively. The characteristics of 600 airborne disease spores were extracted. The two-dimensional relative light-intensity distribution of diffraction–polarization fingerprint images of three spores are shown in Figure 7, Figure 8 and Figure 9, respectively.

The unique relative light-intensity distribution in the directions of 0°, 45°, 90° and 135° may be observed for each airborne disease spore, as depicted in Figure 7, Figure 8 and Figure 9. This phenomenon occurs due to the obstruction of certain light source information by polarized pictures at varying angles due to the polarizer’s influence [17,18,19]. Furthermore, the relative light-intensity distribution values of the diffraction–polarization fingerprint images for three types of airborne disease spores deviate significantly from those documented in existing literature [16]. The reasons are mainly due to the following two aspects: firstly, the CMOS image sensor of device DYSMT805 was used when collecting the diffraction fingerprint image, while the TRIO5OS-QC polarization camera was used when collecting the diffraction–polarization fingerprint image. The parameters of the different imaging devices are different. Secondly, when collecting the diffraction fingerprint image of airborne disease spores, the glass slide containing the enriched airborne disease spores was placed directly on the photosensitive unit of the CMOS image sensor. There was only one layer of glass between the airborne disease spores and the photosensitive unit of the CMOS image sensor. However, when the polarized camera (model TRIO5OS-QC) was used to collect the diffraction–polarized fingerprint images, the glass slide containing the enriched airborne disease spores was placed on the glass sheet of the polarized camera lens. There was a distance of several millimeters between the photosensitive unit of the polarization camera and the glass sheet on its lens. Thus, the distance between the airborne disease spores and the photosensitive units of the different imaging equipment was different. Although the relative light-intensity distribution values of diffraction fingerprint images of airborne disease spores collected by different imaging equipment are different, it will not affect the identification of airborne disease spores. This is because the fingerprint information of airborne disease spores collected by different imaging equipment is relative. The statistical results of disease spore characteristics are shown in Table 1.

### 3.2. Classification Results of Airborne Disease Spores

**Figure 10:**
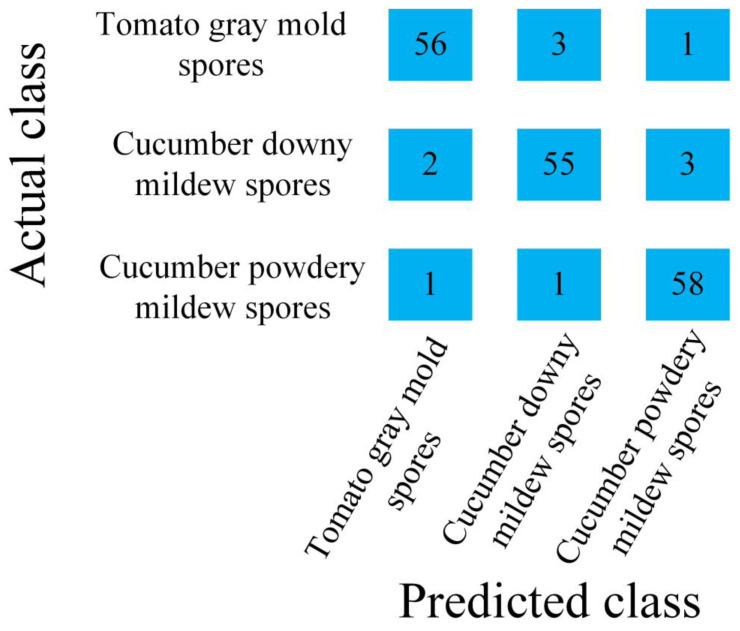
Confusion matrix of SVM classification model.

According to the characteristics data of the airborne disease spore diffraction–polarization fingerprint, six characteristic values of the airborne disease spore diffraction fingerprint at a polarization angle were calculated using Equations (3)–(5). There are 24 values under 4 polarization angles. These values constitute 24 characteristic values of spore diffraction fingerprint images of airborne diseases. The feature values of diffraction fingerprint images were merged to classify airborne disease spores. Therefore, in this study, an SVM algorithm was used to classify three airborne diseases spores according to the eigenvalues of diffraction–polarization fingerprint images. Some 70% of them were randomly selected as the training set and the remaining 30% as the test set. Figure 10 is a confusion matrix of classification results of the SVM classification model for the test set of the airborne disease spore diffraction–polarization fingerprint images.

It can be seen from Figure 10 that the SVM classification model correctly identified 56 diffraction fingerprint images of tomato gray mold spores, 55 diffraction fingerprint images of cucumber downy mildew spores and 58 diffraction fingerprint images of cucumber powdery mildew spores. In the results, the tomato gray mold spores were identified three times as cucumber downy mildew spores and once as powdery mildew. The cucumber downy mildew spores were identified twice as tomato gray mold spores and three times as cucumber powdery mildew spores. One cucumber powdery mildew spore was identified as tomato gray mold spore and one cucumber powdery mildew spore was identified as cucumber downy mildew spore. The identification results of airborne disease spores by SVM classification model are shown in Table 2.

Using Equations (6)–(9), the accuracy, precision, recall and F1-Score of the SVM classification model were evaluated, and the results are shown in Table 3.

As can be seen from Table 3, the accuracy, precision, recall and F1-Score of the SVM classification model for tomato gray mold spores were 96.02%, 94.92%, 93.33% and 94.12%, respectively. The accuracy, precision, recall and F1-Score of the SVM classification model for cucumber downy mildew spores were 94.94%, 93.22%, 91.67% and 92.44%, respectively. The accuracy, precision, recall and F1-Score of the SVM classification model for cucumber powdery mildew spores were 96.57%, 93.55%, 96.67% and 95.08%, respectively. The average recognition accuracy, average recognition precision, average recall rate and average F1-Score of three disease spores by the SVM model were 95.85%, 93.89%, 93.88% and 93.87%, respectively. Therefore, based on the characteristic of the diffraction–polarization fingerprint of disease spores and the SVM algorithm, the disease spores can be well classified.

In addition, compared to the identification results of the airborne disease spore diffraction fingerprint features under visible light (where the average identification accuracy was 92.72%) [16], the average identification accuracy was 95.85%, and the average identification accuracy was improved by 3.13%. Compared to the recognition results based on microscopic image features (where the average recognition accuracy was 94.36%) [32], the average recognition accuracy of the three kinds of airborne disease spores based on diffraction–polarization image features was increased by 1.49%. The results show that the diffraction–polarization image features were effective in the identification of airborne disease spores.

Furthermore, it was compared with the existing literature. For example: Yang et al. [14] utilized a mix of texture and shape features and the decision tree confusion matrix approach to distinguish between rice false smut and blast. The resulting detection accuracy was found to be as high as 94%. Deng et al. [33] used microscopic images of bunt-damaged wheat and image analysis and recognition technology to classify and identify three kinds of wheat diseases, such as *Tilletia caries (DC.) Tul*., *Tilletia indica Mitra* and *Tilletia controversa Kühn.* The results showed that the identification rate of bunt via the SVM method reached 82.9%. Liu et al. [34] focused on the problem of difficult to control marigold black spot. Image processing technology was used to segment the microscopic images of pathogenic spores. The color features, shape features and texture features of pathogenic spores were extracted. Then, principal component analysis (PCA) and BP neural networks were used to identify the spores of Alternaria tagetica without infection and those with infection. The average recognition accuracy reached 98%. Yang et al. [35] studied a method of rice blast identification based on the diffraction fingerprint structure of crop disease spores. The method utilized the light field and texture features of diffraction images. The diffraction identification method based on the diffraction fingerprint texture of crop disease spores can be completed in a few seconds, and the test accuracy was 97.18%. Although the airborne disease spores in this study were different from those in the literature, and the characteristics of the extracted airborne disease spores were different, the results of this paper were similar to those of previous studies. Therefore, the results of this study can be applied elsewhere.

## 4. Conclusions

(1) This study proposed a new classification method of greenhouse crop airborne disease spores based on diffraction–polarization image fingerprint characteristics and machine learning. A diffraction–polarization imaging system was established, and the diffraction fingerprint images of disease spores were taken in polarization directions of 0°, 45°, 90° and 135°. Subsequently, the diffraction–polarization images were processed, wherein the fingerprint features of spore diffraction–polarization images were extracted. Finally, an SVM classification model was established to realize the classification of disease spores.

(2) Six characteristic values of airborne disease spore diffraction fingerprints at a polarization angle were calculated. There were 24 values under 4 polarization angles. The feature values of the diffraction fingerprint images were merged to classify the airborne disease spores. The average recognition accuracy, average recall rate and average F1-Score of three airborne disease spores in greenhouse crops by SVM model were 95.85%, 93.89%, 93.88% and 93.87%, respectively.

In this study, we introduced a method to classify tomato gray mold spores, cucumber downy mildew spores and cucumber powdery mildew spores by using diffraction–polarization image fingerprint characteristics and machine learning. In addition to the above three disease spores, the greenhouse air also contained other kinds of disease spores, pollen, dust and so on. Future research can consider the following two aspects: first, the separation and enrichment methods of micro-particles in the air to achieve the separation and enrichment of different types and sizes of disease spores; second, the diffraction–polarization image fingerprint characteristics of other kinds of disease spores, pollen, dust and other micro-particles, and to establish a corresponding database.

## Figures and Tables

**Table 1:** Statistical results for disease spore characteristics.

Feature		Relative Light-Intensity Distribution Value		
		Cucumber Downy Mildew Spores	Cucumber Powdery Mildew Spores	Tomato Gray Mold Spores
Central value (C)	0°	84–93	67–74	112–119
	45°	87–96	69–78	117–126
	90°	93–104	65–73	109–117
	135°	88–97	68–75	115–124
Wave peak value (P)	0°	111–119	93–108	138–149
	45°	121–132	84–105	147–161
	90°	117–129	91–104	149–158
	135°	112–124	98–112	151–167
Valley value (V)	0°	67–75	41–49	73–85
	45°	70–78	43–51	71–84
	90°	69–82	38–47	78–89
	135°	62–74	40–47	76–87

**Table 2:** Classification results for SVM classification models.

Spore Species	Basic Indicators			
	*TP*	*TN*	*FP*	*FN*
Gray mold spores	56	113	3	4
Downy mildew spores	55	114	4	5
Powdery mildew spores	58	111	4	2

**Table 3:** SVM classification model performance indicators (%).

Spore Species	Performance Index			
	Accuracy	Precision	Recall	F1-Score
Gray mold spores	96.02	94.92	93.33	94.12
Downy mildew spores	94.94	93.22	91.67	92.44
Powdery mildew spores	96.57	93.55	96.67	95.08

## Data Availability

Data are contained within the article.

## References

[1] Gong X., Liu H., Sun J., Gao Y., Zhang H. (2019). Comparison of Shuttleworth-Wallace model and dual crop coefficient method for estimating evapotranspiration of tomato cultivated in a solar greenhouse. Agric. Water Manag..

[2] Tiwari R.K., Lal M.K., Naga K.C., Kumar R., Chourasia K.N., Subhash S., Kumar D., Sharma S. (2020). Emerging roles of melatonin in mitigating abiotic and biotic stresses of horticultural crops. Sci. Hortic..

[3] Wang T., Wu G., Chen J., Cui P., Chen Z., Yan Y., Zhang Y., Li M., Niu D., Li B. (2017). Integration of solar technology to modern greenhouse in China: Current status, challenges and prospect. Renew. Sustain. Energy Rev..

[4] Bandamaravuri K.B., Nayak A.K., Bandamaravuri A.S., Samad A. (2020). Simultaneous detection of downy mildew and powdery mildew pathogens on *Cucumis sativus* and other cucurbits using duplex-qPCR and HRM analysis. AMB Express.

[5] Zhang C., Li X., Yan H., Ullah I., Zuo Z., Li L., Yu J. (2020). Effects of irrigation quantity and biochar on soil physical properties, growth characteristics, yield and quality of greenhouse tomato. Agric. Water Manag..

[6] Zhao H., Zhao Y., Hu J. (2020). Dissipation, residues and risk assessment of pyraclostrobin and picoxystrobin in cucumber under field conditions. J. Sci. Food Agric..

[7] Miao Y., Luo X., Gao X., Wang W., Li B., Hou L. (2020). Exogenous salicylic acid alleviates salt stress by improving leaf photosynthesis and root system architecture in cucumber seedlings. Sci. Hortic..

[8] Kim T.Y., Ku H., Lee S.-Y. (2020). Crop Enhancement of Cucumber Plants under Heat Stress by Shungite Carbon. Int. J. Mol. Sci..

[9] Xie C., Yang C., He Y. (2017). Hyperspectral imaging for classification of healthy and gray mold diseased tomato leaves with different infection severities. Comput. Electron. Agric..

[10] Wallace E.C., D’Arcangelo K.N., Quesada-Ocampo L.M. (2020). Population Analyses Reveal Two Host-Adapted Clades of *Pseudoperonospora cubensis*, the Causal Agent of Cucurbit Downy Mildew, on Commercial and Wild Cucurbits. Phytopathology.

[11] Sireesha Y., Velazhahan R. (2017). Rapid and specific detection of Peronosclerospora sorghi in maize seeds by conventional and real-time PCR. Eur. J. Plant Pathol..

[12] Lei Y., Yao Z., He D. (2018). Automatic detection and counting of urediniospores of Puccinia striiformis f. sp. tritici using spore traps and image processing. Sci. Rep..

[13] Lei Y., Yao Z.F., He D.J. (2018). Design and Experiment of Micro-image Remote Acquisition System of Uredinispores of Puccinia striiformis f.sp. Tritici. Trans. Chin. Soc. Agric. Mach..

[14] Yang N., Qian Y., El-Mesery H.S., Zhang R., Wang A., Tang J. (2019). Rapid detection of rice disease using microscopy image identification based on the synergistic judgment of texture and shape features and decision tree–confusion matrix method. J. Sci. Food Agric..

[15] Luo Y., Joung H.-A., Esparza S., Rao J., Garner O., Ozcan A. (2021). Quantitative particle agglutination assay for point-of-care testing using mobile holographic imaging and deep learning. Lab Chip.

[16] Wang Y., Mao H., Xu G., Zhang X., Zhang Y. (2022). A Rapid Detection Method for Fungal Spores from Greenhouse Crops Based on CMOS Image Sensors and Diffraction Fingerprint Feature Processing. J. Fungi.

[17] Yang L., Chen W., Bi P., Tang H., Zhang F., Wang Z. (2022). Improving vegetation segmentation with shadow effects based on double input networks using polarization images. Comput. Electron. Agric..

[18] Meng J., Ren W., Yu R., Wu D., Zhang R., Xie Y., Wang J. (2023). Contrast enhanced color polarization image fusion. Optik.

[19] Zhao Y., Reda M., Feng K., Zhang P., Cheng G., Ren Z., Kong S.G., Su S., Huang H., Zang J. (2020). Detecting Giant Cell Tumor of Bone Lesions Using Mueller Matrix Polarization Microscopic Imaging and Multi-Parameters Fusion Network. IEEE Sens. J..

[20] Jiang W., Lu J.Q., Yang L.V., Sa Y., Feng Y., Ding J., Hu X.-H. (2015). Comparison study of distinguishing cancerous and normal prostate epithelial cells by confocal and polarization diffraction imaging. J. Biomed. Opt..

[21] Eskandari S., Khoshgoftarmanesh A.H., Sharifnabi B. (2017). The Effect of Foliar-Applied Manganese in Mineral and Complex Forms with Amino Acids on Certain Defense Mechanisms of Cucumber (*Cucumis sativus* L.) Against Powdery Mildew. J. Plant Growth Regul..

[22] Jia Z.M., Liu F., Mu W., Wei G., Liu Y.L. (2006). Study on the inoculation and fungicide sensitivity assay method of Sphaerotheca on cucumber. Acta Phytophylacica Sin..

[23] Gül E., Karakaya A., Ergül A. (2023). Determination of the frequency and virulence of some *Botrytis cinerea* isolates and a new *Botrytis prunorum* cryptic species causing gray mold disease on greenhouse tomatoes. Plant Pathol..

[24] Yu Z., Li Y., Deng L., Luo B., Wu P., Geng D. (2023). A high-performance cell-phone based polarized microscope for malaria diagnosis. J. Biophotonics.

[25] Xiao W., Xin L., Cao R., Wu X., Tian R., Che L., Sun L., Ferraro P., Pan F. (2021). Sensing morphogenesis of bone cells under microfluidic shear stress by holographic microscopy and automatic aberration compensation with deep learning. Lab Chip.

[26] Prasobhkumar P.P., Venukumar A., Francis C.R., Gorthi S.S. (2021). Pebrine diagnosis using quantitative phase imaging and machine learning. J. Biophotonics.

[27] Lin Y.-H., Huang H.-H., Wang Y.-J., Hsieh H.-A., Chen P.-L. (2022). Image-based polarization detection and material recognition. Opt. Express.

[28] Su Y.Y., Jin Z.X., Cui Y.X. (2021). Effects of two elicitors on disease resistance and rhizosphere bacterial community of tomato. J. Biol..

[29] Zhang J., Pan C., Liu S., Kou Y., Tang J., Wang Y., Yang N., Huang R. (2021). Crop Disease Source Location and Monitoring System Based on Diffractive Light Identification Airborne Spore Sensor Network. IEEE Internet Things J..

[30] Vijayaragavan P., Ponnusamy R., Aramudhan M. (2020). An optimal support vector machine based classification model for sentimental analysis of online product reviews. Future Gener. Comput. Syst..

[31] Almoujahed M.B., Rangarajan A.K., Whetton R.L., Vincke D., Eylenbosch D., Vermeulen P., Mouazen A.M. (2022). Detection of fusarium head blight in wheat under field conditions using a hyperspectral camera and machine learning. Comput. Electron. Agric..

[32] Wang Y., Du X., Ma G., Liu Y., Wang B., Mao H. (2020). Classification Methods for Airborne Disease Spores from Greenhouse Crops Based on Multifeature Fusion. Appl. Sci..

[33] Deng J.Z., Li M., Yuan Z.B., Jin J., Huang H.S. (2012). Feature extraction and classification of Tilletia diseases based on image recognition. Trans. CSAE.

[34] Liu H., Ji R.H., Qi L.J., Ma W., Gao C.H. (2015). Spores of marigold black spot identification based on PCA and BP neural network. J. Chian Agric. Univ..

[35] Yang N., Yu J., Wang A., Tang J., Zhang R., Xie L., Shu F., Kwabena O.P. (2020). A rapid rice blast detection and identification method based on crop disease spores’ diffraction fingerprint texture. J. Sci. Food Agric..

